# Acute effect of precise manual tasks on physiological tremor of both upper limbs in young and elderly people

**DOI:** 10.13075/ijomeh.1896.02647

**Published:** 2026

**Authors:** Joanna Mazur-Różycka, Jan Gajewski

**Affiliations:** 1 Central Institute For Labour Protection – National Research Institute (CIOP-PIB), Department of Ergonomics, Warsaw, Poland; 2 Józef Piłsudski University of Physical Education, Faculty of Physical Education, Warsaw, Poland

**Keywords:** fatigue, age, gender, physiological tremor, upper limb, manual work

## Abstract

**Objectives:**

The aim of the study was to assess physiological tremor as a result of fatigue related to manual activities, taking into account age, gender and upper limb (dominant and non-dominant).

**Material and Methods:**

The study included participants from 2 age groups, 25–35 years and 55–65 years. Work fatigue was induced during a 3-hour (180 min) 2-handed upper limb control task which consisted of 3 stages. The first stage lasted 30 min, the second stage – 60 min (90 min from the beginning of the effort) and the third stage – 90 min (180 min from the beginning of the effort). Measurements of physiological tremor were performed before exercise and at 30 min, 90 min and 180 min of exercise duration. An acceleration measurement set-up for both upper limbs was used to collect tremor time courses. The power spectral density functions of the tremor signals were analyzed.

**Results:**

Amplitude indicators from the frequency range 1–5 Hz and 8–14 Hz were extracted for analysis. The age of the subjects did not significantly affect the parameters of physiological tremor. Amplitude indicators from lower (1–5 Hz) and higher (8–14 Hz) frequencies take lower average values for women. For both indicators, a significant difference was observed in the subsequent measurements. The mean values of both indicators for the non-dominant limb were significantly higher.

**Conclusions:**

Physiological tremor measurement carried out simultaneously for both upper limbs to analyze muscle fatigue during repetitive work can improve the efficiency of assessing the occurrence of fatigue on the job and increase the efficiency of assessing adaptive capabilities.

## Highlights

- The measurement of both upper limbs can improve the efficiency of fatigue assessment.
- Age did not significantly affect the parameters of physiological tremor.
- The waveforms of the power spectral density functions for both genders are characterized by similar shape.

## INTRODUCTION

Physiological tremor is defined as involuntary oscillations of individual parts of the body of healthy people resulting from the interaction of mechanical and nervous factors [1]. Tremor itself does not necessarily have a direct effect on work performance, but workers may find it difficult to coordinate movements and maintain the required level of precision as a result of fatigue, which can lead to mistakes and accidents, as well as an increase in musculoskeletal complaints.

Repetitive movements cause muscle fatigue [2], which has been identified as a risk factor for the development of musculoskeletal disorders [3,4]. Repetitive movements of the upper extremities are regularly performed in a variety of occupations (e.g., manufacturing, assembly line work, services), as well as in activities of daily living. Workers who perform low-stress but highly repetitive tasks (hairdressers or dentists) are more than twice as likely to develop musculoskeletal disorders as workers who perform varied and mobile tasks [5]. Consequently, assessing muscle fatigue in the workplace is crucial for improving preventive health care in workers performing low-load repetitive tasks [6].

Additional stresses occurring in the workplace associated with repetitive work may also be related to the precision of activity performance. It seems that the reduction in the precise performance of activities may be influenced by weakened muscle strength, reduced motor skills and increased muscle tremor, which, as a symptom accompanying fatigue, can be taken as information about its intensity. It has been shown that tremor amplitude varies strongly between individuals [7], while age as well as gender may act on physiological tremor as more indeterminate and indirect determinants [8]. However, the results of studies devoted to the issues of how age and gender affect the parameters of physiological muscle tremor have led to inconclusive results. In the world literature, there are works both those in which the relationship exists [9] and those in which the relationship is absent [10].

Studies of muscle tremor during manual activities requiring precision performing the same tasks may allow a more accurate determination of the effect of muscle fatigue on physiological tremor characteristics in individuals of different ages. In literature studies, the effect of gender on physiological tremor parameters was not clear. Some studies have found gender differences in physiological muscle tremor; however, their results were not definitive [5,10]. At work involving manual activities requiring high precision (e.g., the work of a surgeon, a nurse), the efficiency of adaptive mechanisms and resistance to fatigue play an important role. According to Gandevia [12], muscular fatigue is any exercise-induced decrease in the ability of muscles to develop strength or power. Fatigue is considered a superposition of 2 separate processes. The first relates to changes in the muscle itself (e.g., accumulation of metabolic end product or reduction in the efficiency/effectiveness of neuromuscular conduction) and is defined as peripheral fatigue, while the second process involves central nervous system structures (e.g., a decrease in the frequency of motoneuron discharges) and is understood as central fatigue [13]. Note that both of these fatigue processes occur together.

Among the types of fatigue, it is also important to remember the occurrence of mental fatigue, which arises due to prolonged periods of cognitive activity [14] and can be characterized by feelings of “fatigue” and “reduced propensity to expend energy.” The effect of physical fatigue on changes in tremor has been repeatedly addressed in the literature in both biomechanics and neurophysiology [15]. However, these papers were concerned with typical physical exertions associated with sports, i.e., long intense [16] or short maximal [17]. To the authors' knowledge, there are no literature reports on fatigue from performing monotypic manual work requiring precision on physiological tremor parameters. While investigating the effects of performing such work on tremor could provide data to develop methodologies for assessing such fatigue in a non-invasive manner. In the literature, works on the effect of muscle fatigue on physiological tremor parameters usually concern the dominant (D) upper limb [18]. The study of both the D and non-dominant (ND) upper limb, taking into account the age of the subjects and the performance of 2-handed coordination tasks, is a novel study that provides a broad overview of the research on the effect of fatigue induced by the performance of precise manual activities on the characteristics of physiological tremor.

The purpose of the study was to assess changes of physiological tremor as a result of fatigue related to manual activities, taking into account age, gender and upper limb (D and ND).

## MATERIAL AND METHODS

### Participants

The study included 80 volunteers belonging to 2 age groups of 25–35 years and 55–65 years. Members of the younger group were already at an age where changes related to development and growth are mostly stabilized. At the same time, they do not show any significant effects of ageing. Members of the older group represent middle and late adulthood, in which ageing processes (e.g., decline in motor function, increased neural noise, reduced tissue elasticity) begin to have measurable effects. However, they are not at an age at which various disruptive pathologies are more likely to occur. Comparing these 2 groups provides a good contrast: enough difference to detect the effects of age, but not so much that diseases characteristic of the elderly would overly distort the results. This approach allows the detected effects to be generalized to the working population.

Many precise tasks are performed by people in both age groups, so the results are relevant to occupational health. The comparison between the younger and older groups reflects this developmental change well.

The study group consisted of women and men from Poland (Caucasian). There were 20 women and 20 men in each age study group. The inclusion criteria included age of volunteers. The exclusion criteria included neurological and orthopedic conditions and musculoskeletal injuries. Due to the lack of complete measurement data, 3 subjects (1 woman and 2 men aged 55–65 years) were excluded from the analysis. The final analysis included 77 participants.

The experimental-study was conducted in Warsaw (Poland) at the Central Institute for Labor Protection – National Research Institute in the Biomechanics Laboratory of the Department of Ergonomics in 2020–2022. Approval for the study was granted by the Bioethics Committee at Cardinal Stefan Wyszynski University in Warsaw (KEiB-06/2020).

Before taking the measurements, each subject was informed in detail about the purpose, scheme and method of the study. The subjects signed a written informed consent to join the study, fully aware that they could withdraw from the study at any time without incurring consequences.

The D limb is the limb that a person uses on a daily basis (i.e., to write, eat, throw, etc.). In the group of women aged 25–35 years and in the group of men aged 55–65 years, all participants were right-handed. In the group of women aged 55–65 years and in the group of men aged 25–35 years, there was 1 left-handed person in each group. Table 1 shows the detailed characteristics of the study subjects.

**Table 1.** Descriptive characteristics for all participants in the study among 77 Polish adults, Poland, 2020–2022

Variable	Participants (N = 77)			
	women (N = 39)		men (N = 38)	
	55–65 years (N = 19)	25–35 years (N = 20)	55–65 years (N = 18)	25–35 years (N = 20)
Age [years] (M±SD)	59.24±2.85	30.03±3.81	60.42±3.29	28.80±3.38
Body weight [kg] (M±SD)	71.91±13.09	65.00±12.50	88.44±8.30	86.05±14.63
Body height [cm] (M±SD)	164.95±5.90	167.00±5.84	177.11±6.08	180.30±8.60

### Experimental design

The study was conducted according to an established scheme. The subjects embarked on a 180-minute test, which was interrupted at 30 min and 90 min of exercise. Immediately after coming to the laboratory, the subjects were obliged to rest briefly for about 5–10 min. Then two 32-second measurements of physiological muscle tremor were taken using the accelerometric method for the right and left upper limbs with a 10-minute interval. The second measurement was taken for analysis due to the possibility of increased tremor in the first measurement as a result of a stressful situation. After completing the tremor measurements, the subjects performed a 30-minute control test on the upper limb bimanual coordination tasks stand, after which the physiological upper limb tremor was measured again. Subsequently, the subjects performed 2 more control tests lasting 60 min (2 × 30 min with no break) and 90 min (3 × 30 min with no break). Physiological tremor measurements were taken before exercise and at 30 min, 90 min and 180 min of exercise duration.

### Upper limb control station

During the course of the study, the subjects were asked to complete a series of tasks on the computer, which involved controlling cursors along specific tracks. The control was performed using 2 stationary rods – 1 per hand [19]. Each stick was connected to 2 sensors that measure the moment of force in axes perpendicular to each other. The position of the cursor on the screen was proportional to the moment of force exerted by the test subject on the rod in a given direction [19]. The author-developed control task consisted of 14 tests arranged using a specialized measurement program that is part of the test bench. The execution of the control tasks began and ended with the measurement of the maximum force torque exerted on the control sensors. The measurement of maximum moments was recorded in 4 directions (forward, backward, left, right) and was performed for each hand separately. The measurement was carried out at the beginning and at the end of the training session. The initial measurement was also used to determine control ranges for subsequent tasks. In the tasks, object following is implemented, which involves following a moving object that has a defined starting point, trajectory and end point, or shape tracking. The movement of the marker is realized by exerting a force under static conditions on 2 sensors separately for the left and right upper limbs. Tasks vary in terms of symmetry, complexity, but also the speed of the marker's movement. One control task consisted of immediately consecutive tests. The entirety of 1 control task lasted 30 min. The test subject had to perform a total of 6 such single tasks for a total of 180 min.

### Physiological tremor

The accelerometric method was used to study tremor. The waveforms of the tested signals were recorded using the ZPT-2R Acceleration Measurement Kit (Zbigniew Staniak JBA, Warsaw, Poland) which is designed to measure and analyze the acceleration of physiological tremor in the upper limbs. The kit contains 2 acceleration recorders mounted on 1-kilogram weights made in the form of a flat disc. During measurements of upper limb tremor, the subjects were in a sitting position having their torso and elbow joint supported against a wall. The recorder with a built-in accelerometer was placed on a 1-kilogram weight resting on the subject's hand, held horizontally by the subject in as still a position as possible. Physiological tremor tests were performed simultaneously for the right and left upper limbs. The measurement lasted 32 s. The signals were sampled at the frequency of 200 Hz.

The handling of the measurements and analysis of the measurement results was carried out with specialized TDA1v0 software (Zbigniew Staniak JBA, Warsaw, Poland). The software provides analysis of the physiological tremor spectrum based on the fast Fourier transform (FFT) procedure.

The resultant power density function of the tremor signal (power spectral density – PSD) was calculated as the average of 9 overlapping parts of the measured signal (according to Welch's procedure) [16].

The indices describing the power and frequency of the tremor signal were calculated for each participant based on the PSD function expressed in mm^2^/s^3^. The following variables were defined for the further analysis:

– log amplitude indicator defined as an average of logarithms of power components from f_1_ to f_2_ range:
(1) $$\text{L}({\text{f}}_{1},{\text{f}}_{2})=\frac{1}{{\text{f}}_{2}-{\text{f}}_{1}}\int_{{\text{f}}_{1}}^{{\text{f}}_{2}}\;\text{ln}\text{PSD}(\text{f})\text{df}$$
  where:
  f_1_, f_2_ – the boundaries of the frequency band.
  L(f_1_, f_2_) – log amplitude indicator of frequency band between f_1_ and f_2_,
  PSD(f) – power spectral density function,
– mean frequency of power components from f_1_ to f_2_ range defined as:
(2) $$\text{F}({\text{f}}_{1},{\text{f}}_{2})=\frac{\int_{{\text{f}}_{1}}^{{\text{f}}_{2}}\;\text{f}\cdot \text{PSD}(\text{f})\text{ds}}{\int_{{\text{f}}_{1}}^{{\text{f}}_{2}}\;\text{PSD}(\text{f})\text{df}}$$

### Statistical analysis

Statistical analysis was performed using Statistica 14.0 software. The normality of distributions of variables describing the strength and frequency of physiological tremor (dependent variables) was confirmed using the Shapiro-Wilk test. Analysis of variance (ANOVA) was used to assess differences in the mean values of the studied variables. The ANOVA design included 2 fixed factors:

– gender (men, women),
– group (younger, older),

as well as 2 repeated factors:

– measurement (before the test, after 30 min, after 90 min, after 180 min),
– hand (D, ND).

Sphericity was tested using the Mauchly test. If the sphericity condition was not met, the probabilities were corrected using the Greenhouse-Geisser correction. The minimum sample size for the interaction of the fixed factor and fourfold repetition of the measurement was estimated using the G^*^Power program at 50 cases. The assumptions were α = 0.05, mean effect size η^2^ = 0.06, and test power 1 – β = 0.95. Detailed comparisons for statistically significant effects were made using the Tukey *post hoc* test. Effect sizes were determined for the analysis of variance based on the partial value of η^2^. Values <0.06 were considered small, 0.06–0.14 – moderate, and >0.14 – large. A significance level of α = 0.05 was assumed.

## RESULTS

**Figure 1.**
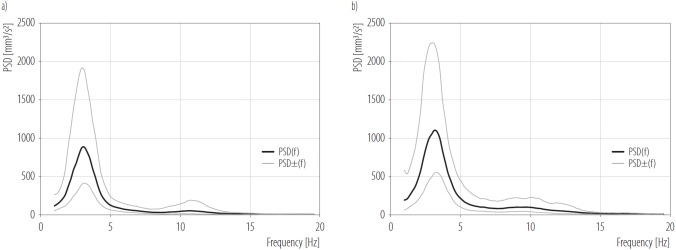
Power spectrum density function of the dominant forearm tremor for young a) female (N = 20) and b) male (N = 20) in the study among 77 Polish adults, Poland, 2020–2022

The PSD function waveforms of men and women are characterized by similarity of shape – they show a correspondence of frequencies for which maxima occur and similar proportions of individual components (Figure 1). Due to the characteristics of the waveforms of the power spectral function of physiological tremor, results from the frequency range of 1–5 Hz and 8–14 Hz were extracted for analysis.

PSD – power spectral density; PSD(f) – average power density function; PSD±(f) – frequency series corresponding to standard deviations of the power density function.

Table 2 show the mean (M) ± standard deviation (SD) values of the indicators describing the amplitude of the 1–5 Hz and 8–12 Hz components of tremor of the ND and D limb, for groups of men and women aged 25–35 years and 55–65 years. For both the index describing the tremor amplitude of the low-frequency (L1–5) and high-frequency (L8–14) components, there was a significant effect of the gender factor. These indicators take smaller mean values for women. The effect of measurement was also significant for both indices (for L1–5 and L8–14). Both indicators took the highest values in the second measurement (Table 3). Tukey's *post hoc* test showed that for L1–5, the mean of the second measurement was greater than the mean of the first measurement (p = 0.0089), and for L8–14, the mean of the second measurement was greater than the mean of the third measurement (p = 0.0500). The remaining pairwise comparison tests were not statistically significant. The mean values of both indicators were also significantly higher for the ND limb. For none of the indicators did the group effect related to the subjects' age occur significantly (p > 0.05).

**Table 2.** Means and standard deviations of physiological tremor variables obtained in consecutive measurements for the non-dominant (ND) and dominant (D) upper limb in the study among 77 Polish adults, Poland, 2020–2022

Variable	Participants (N = 77)			
	women (N = 39)		men (N = 38)	
	55–65 years (N = 19)	25–35 years (N = 20)	55–65 years (N = 18)	25–35 years (N = 20)
L1–5 index (M±SD)				
hand non-dominant				
pre-test	5.784±0.506	5.683±0.717	5.949±0.638	6.162±0.712
after 30 min	5.652±0.551	5.922±0.800	6.209±0.601	6.388±0.644
after 90 min	5.719±0.409	5.793±0.658	6.144±0.746	6.417±0.632
after 180 min	5.659±0.506	5.738±0.715	6.082±0.769	6.457±0.577
hand dominant				
pre-test	5.836±0.479	5.903±0.640	6.033±0.408	6.25±0.659
after 30 min	5.704±0.485	6.205±0.771	6.421±0.551	6.326±0.563
after 90 min	5.73±0.286	5.968±0.632	6.21±0.554	6.288±0.541
after 180 min	5.715±0.415	5.958±0.585	6.241±0.366	6.388±0.493
L8–14 index (M±SD)				
hand non-dominant				
pre-test	3.277±0.64	3.479±0.646	3.800±0.697	3.902±0.541
after 30 min	3.367±0.717	3.813±0.869	3.957±0.607	4.085±0.475
after 90 min	3.253±0.587	3.643±0.807	3.638±0.562	4.153±0.577
after 180 min	3.326±0.775	3.539±0.69	3.747±0.680	4.107±0.548
hand dominant				
pre-test	3.539±0.801	3.536±0.932	3.999±0.627	4.199±0.692
after 30 min	3.422±0.676	3.832±1.005	4.109±0.720	4.102±0.557
after 90 min	3.346±0.794	3.517±0.867	3.915±0.633	4.127±0.587
after 180 min	3.355±0.705	3.55±0.938	3.847±0.634	4.200±0.740
F1–5 frequency (M±SD)				
hand non-dominant				
pre-test	3.050±0.197	3.029±0.191	3.170±0.237	2.961±0.244
after 30 min	3.038±0.257	3.082±0.226	3.030±0.248	2.945±0.273
after 90 min	3.023±0.302	3.006±0.193	3.050±0.268	2.991±0.178
after 180 min	3.049±0.268	3.034±0.191	2.933±0.276	2.961±0.244
hand dominant				
pre-test	3.005±0.245	3.041±0.200	3.108±0.210	3.060±0.194
after 30 min	3.065±0.298	3.066±0.232	3.039±0.323	3.009±0.215
after 90 min	3.006±0.308	3.034±0.215	3.005±0.268	3.000±0.264
after 180 min	3.031±0.336	3.046±0.190	2.942±0.262	2.958±0.222
F8–14 frequency (M±SD)				
hand non-dominant				
pre-test	10.468±0.366	10.430±0.358	10.634±0.415	10.430±0.358
after 30 min	10.613±0.400	10.312±0.287	10.611±0.334	10.312±0.287
after 90 min	10.602±0.425	10.333±0.361	10.637±0.375	10.333±0.361
after 180 min	10.560±0.451	10.312±0.388	10.692±0.347	10.312±0.388
hand dominant				
pre-test	10.538±0.406	10.710±0.385	10.602±0.428	10.426±0.528
after 30 min	10.526±0.461	10.600±0.498	10.553±0.470	10.397±0.461
after 90 min	10.464±0.398	10.568±0.479	10.606±0.398	10.492±0.468
after 180 min	10.409±0.354	10.483±0.369	10.461±0.386	10.419±0.426

**Table 3.** ANOVA effects for physiological tremor variables in the study among 77 Polish adults, Poland, 2020–2022

Effect	F	p	η^2^
L1–5 index			
df 1, 73			
gender	13.89	**0.0004**	0.160
group	2.56	0.1137	0.034
hand	7.65	**0.0072**	0.095
hand × gender	1.67	0.2001	0.022
hand × group	0.00	0.9560	0.000
df 3, 219			
measurement	3.29	**0.0216**	0.043
measurement × gender	1.97	0.1200	0.026
measurement × group	0.55	0.6457	0.008
measurement × hand	2.06	0.1064	0.027
measurement × hand × gender	0.56	0.6434	0.008
measurement × hand × group	0.44	0.7221	0.006
L8–14 index			
df 1, 73			
gender	12.47	**0.0007**	0.146
group	3.51	0.0651	0.046
hand	4.23	**0.0434**	0.055
hand × gender	0.96	0.3293	0.013
hand × group	1.31	0.2570	0.018
df 3, 219			
measurement	3.13	**0.0266**	0.041
measurement × gender	0.30	0.8284	0.004
measurement × group	1.21	0.3057	0.016
measurement × hand	3.92	**0.0094**	0.051
measurement × hand × gender	0.21	0.8873	0.003
measurement × hand × group	1.42	0.2382	0.019
F1–5 frequency			
df 1, 73			
gender	0.292	0.5906	0.004
group	0.070	0.7925	0.001
hand	0.004	0.9508	0.000
hand × gender	0.007	0.9355	0.000
hand × group	0.973	0.3272	0.013
df 3, 219			
measurement	5.542	**0.0011**	0.071
measurement × gender	6.399	**0.0004**	0.081
measurement × group	1.008	0.3900	0.014
measurement × hand	0.754	0.5214	0.010
measurement × hand × gender	0.164	0.9202	0.002
measurement × hand × group	1.513	0.2120	0.020
F8–14 frequency			
df 1, 73			
gender	0.641	0.4261	0.009
group	1.058	0.3072	0.014
hand	0.071	0.7903	0.001
hand × gender	0.204	0.6531	0.003
hand × group	4.876	**0.0304**	0.063
df 3, 219			
measurement	1.163	0.3247	0.016
measurement × gender	0.370	0.7746	0.005
measurement × group	0.826	0.4806	0.011
measurement × hand	1.952	0.1222	0.026
measurement × hand × gender	1.933	0.1251	0.026
measurement × hand × group	1.479	0.2212	0.020

df – degrees of freedom. Bold values are statistically significant. Gender: women, men; group: younger, older; hand: non-dominant, dominant; measurement: pre-test, after 30 min, after 90 min, after 180 min.

The M±SD values of the F1–5 and F8–14 frequencies for the ND and D limb for the groups of men and women aged 25–35 years and 55–65 years are shown in Table 2.

For mean frequencies 1–5 Hz, there was neither a gender effect nor a group effect (Table 3). It was noted, however, that the mean frequency decreased in successive measurements, and this was particularly evident for men (interaction of measurement × gender). For mean frequencies 8–14 Hz, there was neither a gender effect nor a group effect. Interactions of hand × group were observed, however, the *post hoc* test did not indicate any differences.

## DISCUSSION

With regard to the work environment of, for example surgeons, nurses or workers who perform monotypic manual work that requires precision, it is important to maintain proper function of the upper limb neuromotor system, as this can be crucial in preventing accidents at work or mistakes made by workers, and in adjusting the pace of work to the worker's capabilities.

As mentioned in the introduction in work related to manual activities requiring high precision (such as the work of a surgeon, nurse) the efficiency of adaptive mechanisms and resistance to fatigue play an important role. Muscle tremor, whether physiological, pathological or mechanical, is a complex phenomenon that can be influenced by both peripheral mechanisms and activity in several areas of the central nervous system, including the motor cortex (central factors) [20]. For this reason, mental fatigue can affect it [18]. However, there are studies whose results do not provide evidence that mental fatigue affects neuromuscular parameters, which affect, for example, postural tremor or dexterity, measured in single-task conditions [21].

Physical exertion can affect the increase in the amplitude of physiological tremor, and the values of changes in the amplitude and frequency of tremor depend on the duration of exercise and its type. Studies show that strength training [22], increasing intensity exercise [16] as well as resistance exercise [23] significantly increase the amplitude of physiological tremor. Gajewski et al [16], studying changes in physiological tremor during high-intensity exercise, observed that the greatest changes in tremor amplitude were seen in the higher frequency range (10–20 Hz). As a result of fatigue, mainly low-frequency components of several hertz increase in the electromyography signal spectrum, thus decreasing the average frequency of the spectrum [24]. Fatigue-induced changes in tremor amplitude are thought to result from temporal disruption of control mechanisms in the nervous system [25]. Fuglsang-Fredriksen and Ronager [26] suggest that a decrease in the firing frequency of motor units may be a symptom of increasing central fatigue.

This study showed a significant difference in the amplitude of physiological tremor, and both analyzed indices (L1–5 and L8–15) took the highest values in the second measurement. In the case of L1–5, the mean of the second measurement was greater than the mean of the first measurement (p = 0.0089), and in the case of L8–14, the mean of the second measurement was greater than the mean of the third measurement (p = 0.0500). A review of the literature found that tremor amplitude varies strongly between individuals [7]. Age and gender, may act on physiological tremor as more indeterminate and indirect determinants [8]. However, the results of studies devoted to the issues of the influence of age and gender on the parameters of physiological muscle tremor have led to inconclusive results.

In the world literature there are works both in which such a relationship exists and those in which the relationship is absent. In a study conducted in a group of 117 healthy subjects aged 20–94 years, no significant effect of age on tremor frequency was found, while the subjects' gender slightly but significantly changed the range of hand tremor frequency. However, multiple partial correlations revealed that the only direct effect on hand tremor frequency was hand volume indicating that the effect of gender on hand tremor frequency is an indirect effect produced by the much larger hands of men, meaning that the effect of the subjects' gender on tremor frequency represents only an indirect mechanical effect [10].

In contrast, a study in a group of 1158 healthy subjects aged 40–98 years showed that tremor increased with age (p < 0.001), and the results of the conducted test were higher in men than in women (p < 0.001). The characteristics of normal physiological tremor have been shown to be highly dependent on posture and the mechanics of the oscillatory system, indicating strong peripheral resonance mechanisms [10]. Also, Louis et al. [9], in a population-based study involving >2500 healthy adults (18–60 years), confirmed a clear association of physiological tremor severity with age (r = 0.06, p = 0.004). They showed that the spiral score increased with each successive decade (p = 0.002). This score in participants in the highest age group (60 years) was about twice as high as in those in the youngest age group (18–19 years) (p = 0.003). These data suggest that the age-dependent increase in tremor amplitude is not limited to the elderly, but occurs in all adult age groups.

This study showed that the age of the subjects did not significantly affect the parameters of physiological tremor. However, it was shown that the waveforms of the PSD function in men and women are characterized by similar shape – they show a correspondence of frequencies for which maxima occur, as well as similar proportions of individual components. Both for the index describing the tremor amplitude of the low-frequency (L1–5) and high-frequency (L8–14) components, a statistically significant difference was found between men and women. These indices take lower average values for women.

In literature studies, the effect of gender on physiological tremor parameters was not clear. Some studies found gender differences, their results were not definitive [10,11]. In 1 study, no difference in amplitude was observed in physiological tremor [10]. In contrast, other authors showed that men showed greater fluctuations than women [11].

In the literature, work on the effect of muscle fatigue on physiological tremor parameters is only concerned with the D upper limb [18]. And it seems that studies of muscle tremor during manual activities requiring precision performing the same tasks may allow a more accurate determination of the effect of muscle fatigue on physiological tremor characteristics. It has been observed that the amplitude of tremor in the right hand of right-handed people is smaller than in the left hand [10,27]. The authors' own research showed that the averages for the ND limb were significantly higher, both for the values of the index describing the tremor amplitude of the low-frequency (L1–5) and high-frequency (L8–14) components.

The main strength of this study is that it takes into account multiple factors simultaneously. In the literature, studies on the impact of muscle fatigue on physiological tremor usually focus on one upper limb. In this study, tremors in both limbs were taken into account, as the tasks performed required coordination of both hands. At the same time, the age and gender of the subjects was also taken into consideration. All this makes it an innovative study assessing the impact of fatigue caused by performing precise manual tasks on the characteristics of physiological tremor. However, certain limitations should also be taken into account when interpreting these results. Although the protocol for inducing fatigue using the upper limb control station involving both limbs was carefully designed, the complex and monotonous nature of these activities may not be replicable in real-life conditions. The device used also enforces a specific limb position, which may be unnatural for some individuals and cause greater fatigue. Coordination difficulties (especially in the initial phase of the test) may also cause increased stress affecting tremor. These limitations point to several important directions for future research.

## CONCLUSIONS

The results of the study show how the effects of fatigue translate into changes in physiological tremor. In professions requiring high manual precision (e.g., surgeons, nurses, technical workers), even a slight increase in tremor amplitude caused by fatigue can translate into a higher risk of errors or accidents. Fatigue has been shown to significantly increase the amplitude of physiological tremor, particularly in the low-frequency (L1–5) and high-frequency (L8–14) components. This supports the hypothesis that central and peripheral fatigue mechanisms may interfere with neuromotor control. No association was found between age and tremor parameters, although the literature presents inconsistent results, with some evidence of an age-related increase in tremor amplitude during adulthood. The gender differences observed in this study suggest a slightly lower tremor amplitude in women, which may indirectly reflect anthropometric factors (e.g., upper limb size) rather than fundamental neurophysiological differences.

In conclusion, physiological tremor measurement carried out simultaneously for both upper limbs to analyze muscle fatigue during repetitive work can improve the efficiency of assessing the occurrence of fatigue on the job and increase the efficiency of assessing adaptive capabilities.

Further research should include: investigating the impact of fatigue on physiological tremor parameters in realistic, real-life working conditions, and analyzing tremor in specific occupations (surgery, nursing, manufacturing) where tasks requiring precision and limited physiological tremor are performed.
